# Supplementary material for: Exploring the Replication and Pathogenic Characteristics of Alpha, Delta, and Omicron Variants of SARS-CoV-2
Source: Int J Mol Sci. 2024 Nov 25;25(23):12641. doi: 10.3390/ijms252312641 (PMC11641553; doi:10.3390/ijms252312641)
Supplement: Supplementary file 1 [file ijms-25-12641-s001.zip › ijms-3309322-supplementary.pdf]

Supplementary Information

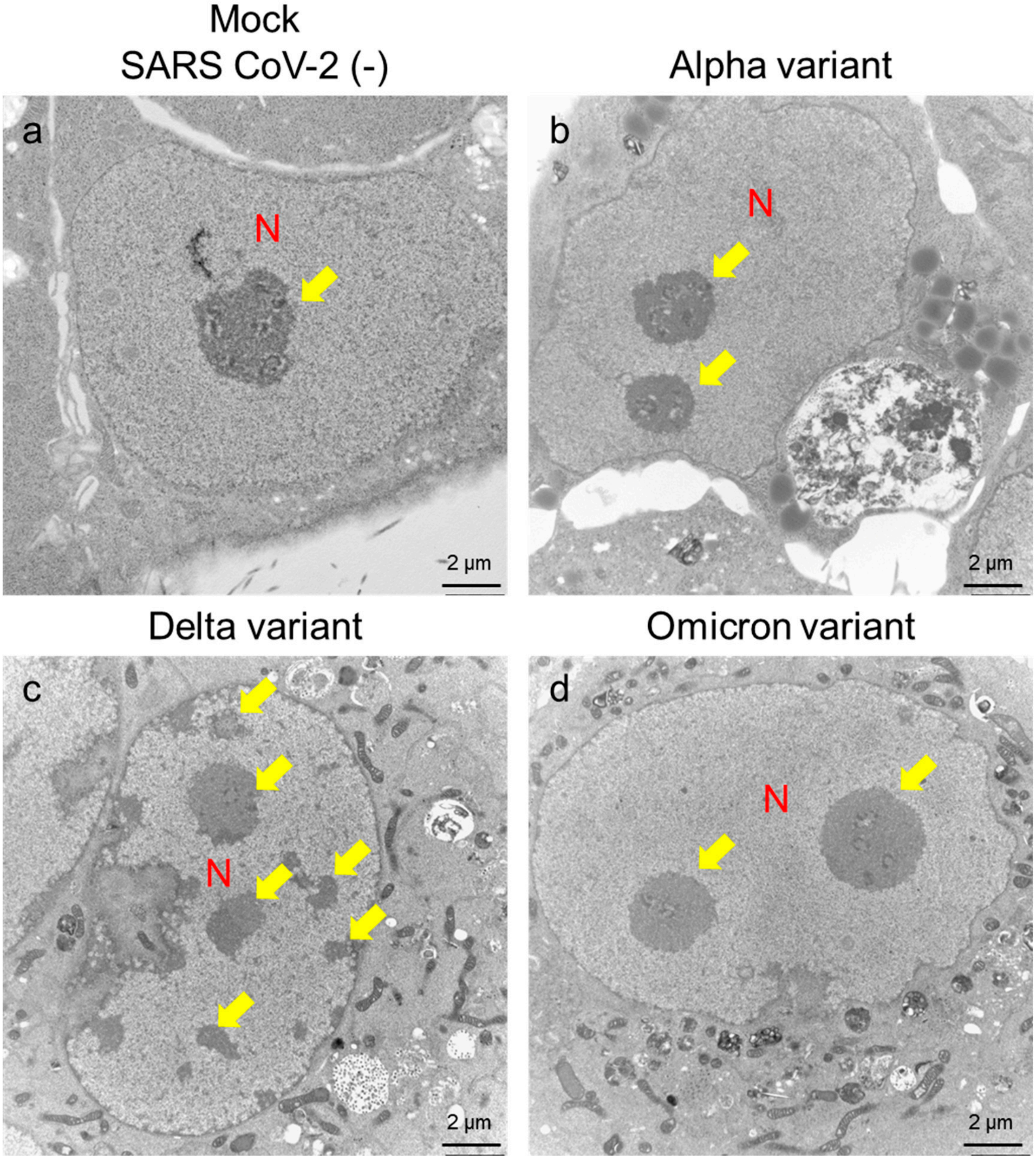

Figure S1. The Delta variant of severe acute respiratory syndrome coronavirus 2 (SARS-CoV-2) formed larger syncytia than the Alpha or Omicron variant in VeroE6/TMPRSS2 cells. (a-d) Representative transmission electron microscopy images indicate the syncytia formation patterns of Alpha, Delta, and Omicron variants of SARS-CoV-2 at 24 hours post-inoculation. Arrows indicate nuclei in mock or SARS-CoV-2-infected cells. N: nucleus.

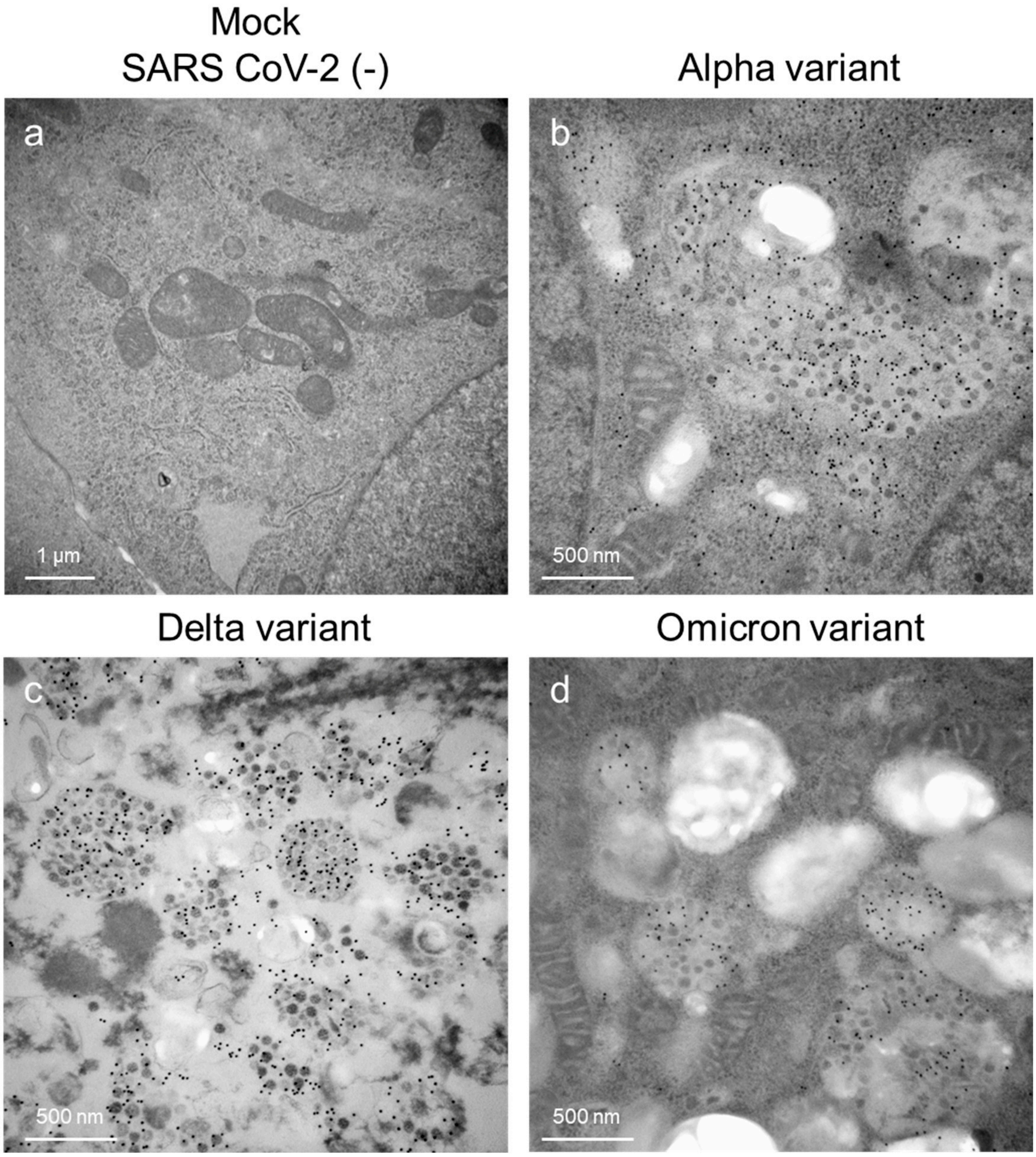

Figure S2. Nucleocapsid (N-protein) of severe acute respiratory syndrome coronavirus 2 (SARS-CoV-2) detected specifically in infected cells. (a-d) Ultrathin sections of mock or infected cells at 48-hour post-inoculation were stained with anti-SARS-CoV-2 N-protein antibody. Representative immunoelectron microscopic images confirmed the immunoreactivities of N-protein of SARS-CoV-2 (gold labelling) in Alpha-, Delta- or Omicron-infected cells but not in uninfected cells.

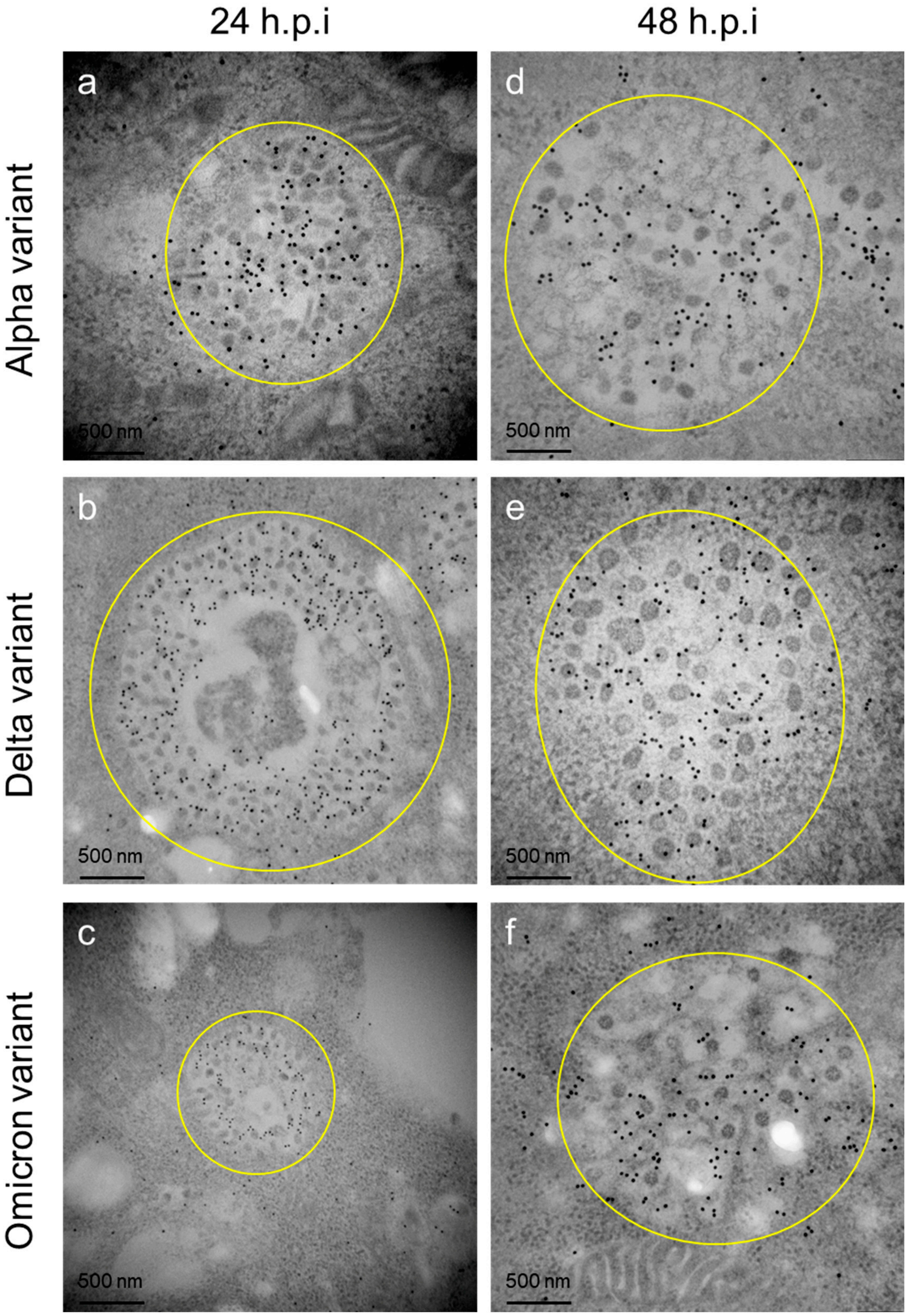

Figure S3. The Delta variant of severe acute respiratory syndrome coronavirus 2 (SARS-CoV-2) showed greater accumulation characteristics of the nucleocapsid (N-

protein) in the VeroE6/TMPRSS2 cells than the Alpha or Omicron variant. (a-f) Ultrathin sections of infected cells at 24-hour post-inoculation (h.p.i.) or 48 h.p.i were stained with anti-SARS-CoV-2 N-protein antibody. Representative immunoelectron microscopic images indicate the accumulation patterns of SARS-CoV-2 N-protein in the intracellular vesicles when cells were infected with the Alpha, Delta, or Omicron variant (indicated by circles).

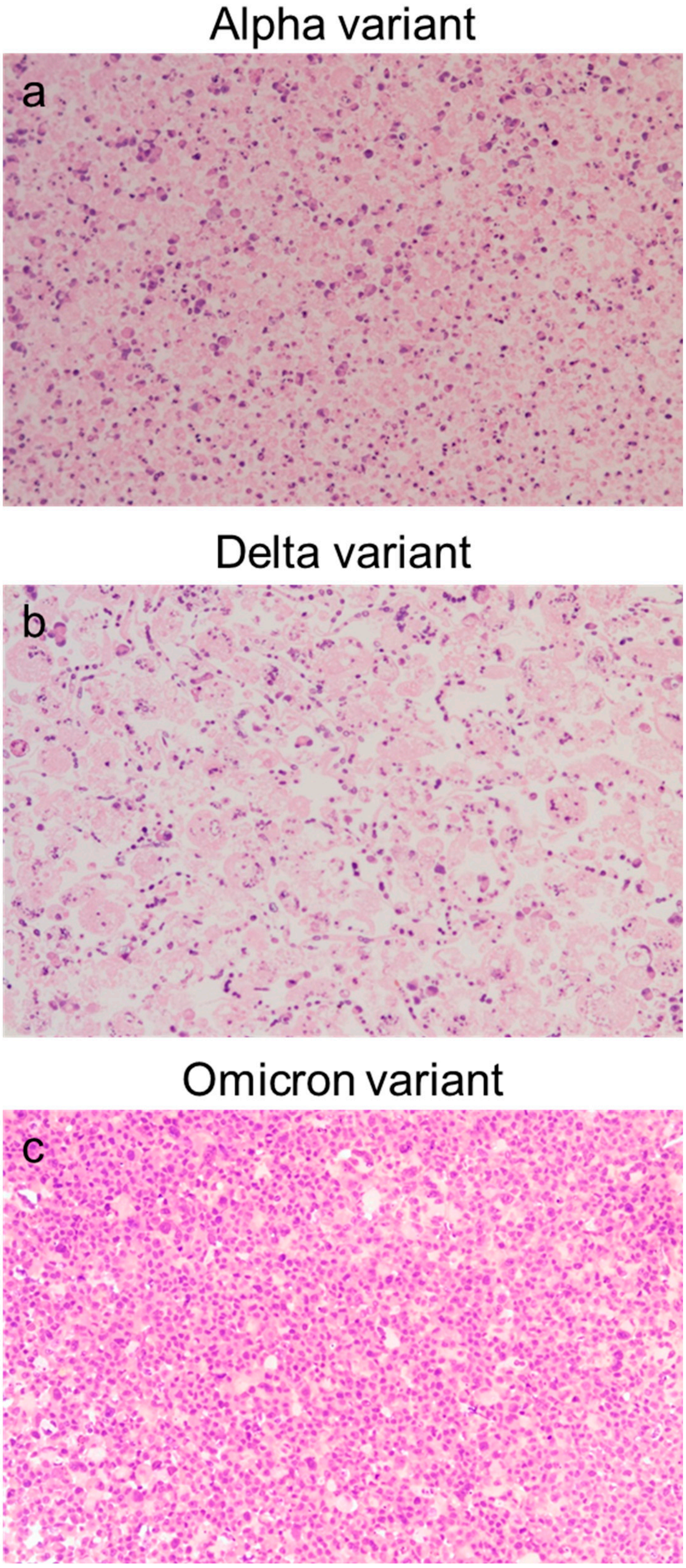
Alpha variant
a
Delta variant
b
Omicron variant
c

Figure S4. Pathological features of major variants of concern of severe acute respiratory syndrome coronavirus 2 in VeroE6/TMPRSS2 cells. (a-c) Sections from Alpha, Delta, and Omicron infected cells at 96 hours post-inoculation were stained with hematoxylin and eosin (H&E).

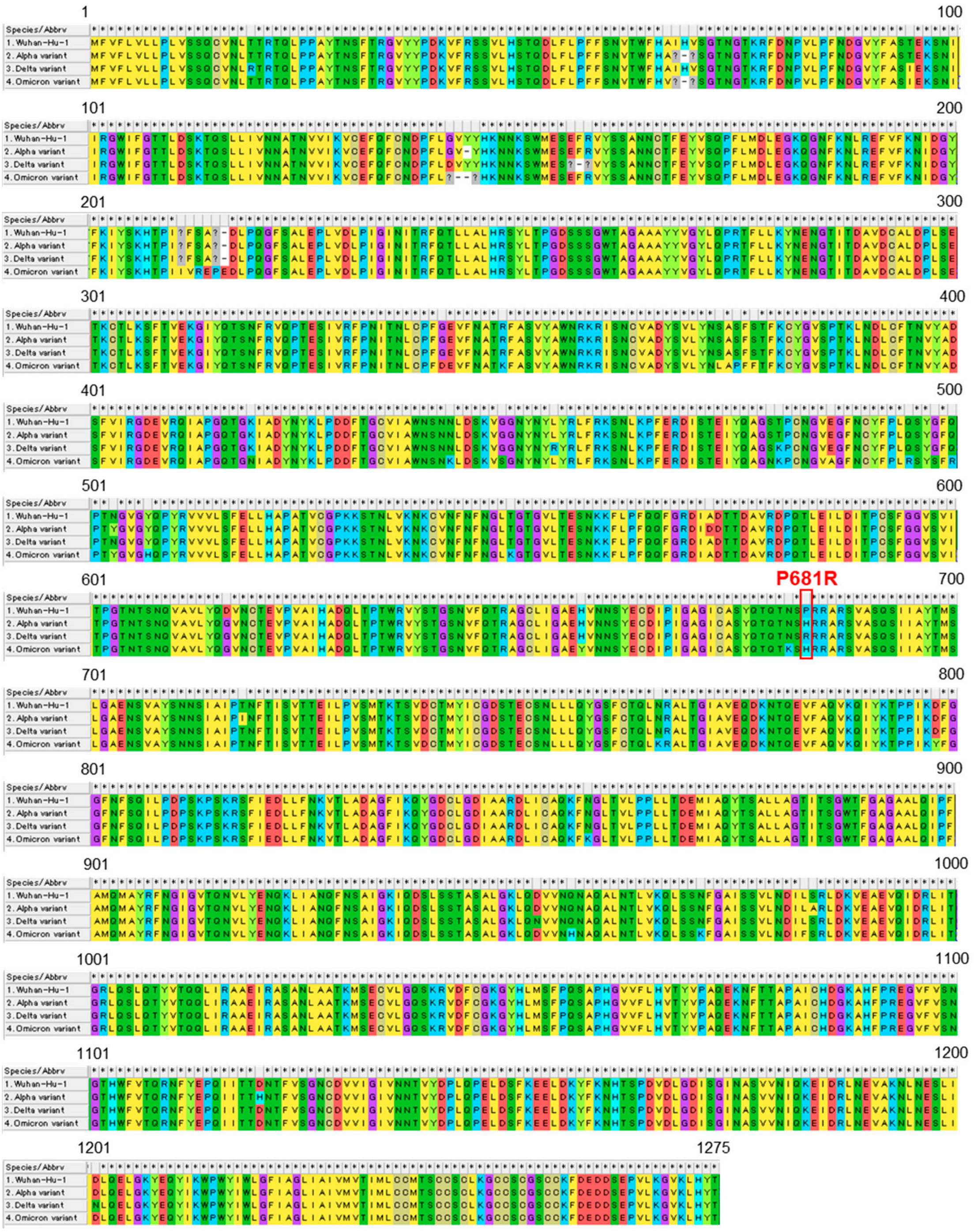

Figure S5. Unique mutations were detected in the spike-protein of Alpha, Delta, and Omicron variants of severe acute respiratory syndrome coronavirus 2 (SARS-CoV-2). Multiple sequence alignment of amino acids between variants and reference (Wuhan Hu-1) type of SARS CoV-2.
